# Editorial: Looking at the Complete Picture: Tackling Broader Factors Important for Advancing the Validity of Preclinical Models in Disease

**DOI:** 10.3389/fnbeh.2022.905327

**Published:** 2022-04-26

**Authors:** Tim Karl, Jess Nithianantharajah, Ulrike Weber-Stadlbauer

**Affiliations:** ^1^School of Medicine, Western Sydney University, Sydney, NSW, Australia; ^2^The Florey Institute of Neuroscience and Mental Health, University of Melbourne, Melbourne, VIC, Australia; ^3^Institute of Pharmacology and Toxicology, University of Zurich-Vetsuisse, Zürich, Switzerland; ^4^Neuroscience Center Zurich, University of Zurich and ETH Zurich, Zürich, Switzerland

**Keywords:** preclinical research, mouse model, validity, animal research, translational value

Experimental research in animal models remains pivotal for expanding our biological understandings into human disease etiology and the development of novel therapies. While they provide controlled model systems with which to address targeted questions, preclinical animal studies often not only ignore the complexity of the human disease they aim to model (Perlman, 2016; Pound and Ritskes-Hoitinga, 2018) but also the sensitivity of animal models and test systems utilized, as well as environmental factors which include experimental test specifics. Indeed, there is mounting evidence that housing conditions, handling procedures and even the sex of the researchers carrying out the experiments can alter the phenotypes of animal models (Crabbe et al., 1999; Hurst and West, 2010; Logge et al., 2014; Sorge et al., 2014).

Therefore, increasing the awareness of researchers in the field to critical factors that can be potential test confounds is important going forward. In line with this notion, awareness of the strong sex bias and disparity in preclinical research (Shansky and Murphy, 2021) with current studies predominantly being undertaken in male animals, is slowly shifting the standard within the field. This has been catalyzed by structural changes within the research system (journals and funding institutions highlighting the need to assess both sexes). Including both male and female animals and robustly assessing sex-dependent effects is essential for translating preclinical work to deliver real-world public health outcomes (Coiro and Pollak, 2019). Reproducibility of research findings also remains a critically important and ongoing issue for scientific research communities, which can be negatively exacerbated by competition within the sector fuelling the pressure to obtain funding and publish. While multiple factors contribute to challenges with reproducibility, the early identification of potential test confounders and inclusion of approaches that address those into experimental design can improve reproducibility. Additionally, greater requirements for rigorous reporting of methodological details in publications as is now required by a growing number of journals, is essential for enhancing transparency and comparability of results obtained across laboratories.

This special topic explores some of the challenging factors still evident in preclinical research with the aim to advance the validity of preclinical models of disease and truly enable translational outcomes for human conditions (Steckler et al., 2015; Pound and Ritskes-Hoitinga, 2018). Articles in this special topic will stimulate considerations of broader variables that impact the translational value of preclinical models for research into human diseases and outline new strategies to help address them.

Varholick et al. shed light on how home cage social hierarchies may impact common behavioral measures. Social dominance status of mice within the home cage are largely ignored in experimental design, analysis and reporting. Conducting a systematic review and meta-analyses of nearly 700 biomedical research studies, the authors found only 20 publications met inclusion criteria due to high heterogeneity in study characteristics and results reported, with little evidence for systematic phenotypic differences between dominant and subordinate male mice. Future studies will need to address the issue of heterogeneity across study designs, and further evaluate these secondary sources of variation. In addition, clinically relevant experimental parameters (e.g., physiological and immunological markers) and sex effects should be considered.

Shepherd et al. assessed how external factors like behavioral testing itself impacts behavioral and neurological phenotypes. Specifically, the team evaluated how training on the touchscreen system, a behavioral tool increasingly used as an approach to improve the translational value of experimental test outcomes for the clinical setting, may influence phenotypes in the *APPswe/PS11E9* transgenic mouse model for Alzheimer's disease. Their results convincingly show that components of the experimental design can directly impact the face validity of established mouse models. The authors conclude that these potential impacts need to be considered when interpreting findings.

The systematic review by Ferland-Beckham et al. utilizes a broad approach to examine the effect of multiple methodological variations on the validity of the single prolonged stress (SPS) paradigm. The team has developed a strategy for the management of SPS test protocol variations considering behavioral coding, statistical approaches and data presentation. Methodological guidelines from an expert panel were generated to provide researchers with a guide for the valid application of SPS in combination with extinction testing to evaluate posttraumatic stress disorder (PTSD)-like phenotypes. Current shortfalls in reporting experimental details (including omitting statistical details, insufficient description of housing conditions, lack of test protocol details) are also discussed. The authors clarify that standardization of laboratory conditions and test protocols will not abolish all phenotypic variation (e.g., because of inter-individual differences), and stress that experimental outcomes need to be testable across a variety of conditions and even species to make the insights gained more relevant to the human condition (Voelkl et al., 2021).

Sil et al. have designed “PEERS” (Platform for the Exchange of Experimental Research Standards), an open-access online tool to enable the identification of experimental factors most likely to impact experimental outcomes, thus should receive particular attention during experimental design, execution, and reporting. A first working prototype using the open field paradigm in rodents provides an initial insight into the practicalities of this platform. The authors' aim is for the platform to foster collaborative exchange and enhance data validity and robustness, as well as the reproducibility of preclinical research.

Finally, Loss et al. provide general insights into how the ever-increasing focus on animal welfare may not only be highly relevant for achieving greater acceptance of preclinical research by the wider community, but also for improving the robustness and validity of animal experimental outcomes—in line with the concept that “*happy animals make better science”* (Poole, 1997; Grimm, 2018). They highlight the development of guidelines for good experimental practices, and that embracing open research practices does not necessarily *assure compliance with the proposed guidelines* (Baker et al., 2014; Hair et al., 2019). One recommendation is to integrate such guidelines into policies related to the award of research funding and acceptance of research articles.

To conclude, preclinical animal models are essential research tools for advancing mechanistic understandings into human disease. There remains, however, mounting awareness and need for greater robustness, replicability, and validity of experimental outcomes in preclinical models. The articles in this special topic highlight the status of the field, challenges that lie ahead and importantly, suggestions for how to improve the validity of preclinical disease models.

## Author Contributions

TK, JN, and UW-S—manuscript writing. All authors contributed to the article and approved the submitted version.

## Conflict of Interest

The authors declare that the research was conducted in the absence of any commercial or financial relationships that could be construed as a potential conflict of interest.

## Publisher's Note

All claims expressed in this article are solely those of the authors and do not necessarily represent those of their affiliated organizations, or those of the publisher, the editors and the reviewers. Any product that may be evaluated in this article, or claim that may be made by its manufacturer, is not guaranteed or endorsed by the publisher.
